# Establishment of Sample-to-Answer Loop-Mediated Isothermal Amplification-Based Nucleic Acid Testing Using the Sampling, Processing, Incubation, Detection and Lateral Flow Immunoassay Platforms

**DOI:** 10.3390/bios14120609

**Published:** 2024-12-13

**Authors:** Lilas Pommiès, Hervé Boutal, David Fras, Hervé Volland

**Affiliations:** 1 CEA, INRAE, Département Médicaments et Technologies pour la Santé (DMTS), Université Paris-Saclay, SPI, 91191 Gif-sur-Yvette, France; lilas.pommies@gmail.com (L.P.); herve.boutal@cea.fr (H.B.); 2 CEA/DRT/LIST/DIN/SIMRI, 91191 Gif-Sur-Yvette, France; david.fras@cea.fr

**Keywords:** sample-to-answer test, POCT, genetic detection

## Abstract

Diagnostics often require specialized equipment and trained personnel in laboratory settings, creating a growing need for point-of-care tests (POCTs). Among the genetic testing methods available, Loop-mediated Isothermal Amplification (LAMP) offers a viable solution for developing genetic POCT due to its compatibility with simplified devices. This study aimed to create a genetic test that integrates all steps from sample processing to analyzing results while minimizing the complexity, handling, equipment, and time required. Several challenges were addressed to achieve this goal: (1) the development of a buffer for bacterial DNA extraction that is compatible with both LAMP and immunochromatographic tests; (2) the adaption of the LAMP protocol for use with the SPID device; and (3) the optimization of the detection protocol for specific test conditions, with a lateral flow immunoassay format selected for its POCT compatibility. Following these developments, the test was validated using *Escherichia coli* (*E. coli*) and non-*E. coli* strains. A portable heating station was also developed to enable amplification without costly equipment. The resulting genetic POCT achieved 100% sensitivity and 85% specificity, with results available in 60 to 75 min. This study demonstrated that our POCT efficiently performs DNA extraction, amplification, and detection for bacterial identification. The test’s simplicity and cost-effectiveness will support its implementation in various settings.

## 1. Introduction

Most diagnostics are performed by trained personnel using specialized instruments in a laboratory setting [1]. The time required to obtain results can vary depending on several factors, including the type of sample being analyzed, the specific test being conducted, and whether the sample needs to be transported from the collection site to a laboratory facility [2]. In recent years, there has been a growing demand for point-of-care testing (POCT) that offers a rapid, simple, and cost-effective diagnosis. POCT is particularly valuable in developing countries where access to healthcare facilities and advanced laboratory equipment may be limited. These tests allow for fast decision-making and can potentially improve patient outcomes by enabling timely interventions.

The World Health Organization (WHO) and the US Food and Drug Administration (FDA) have recognized the importance of POCT and actively support its development. To ensure the quality and effectiveness of these tests, they have established the REASSURED criteria, which define the standards for ideal point-of-care tests. These criteria include real-time connectivity, ease of specimen collection and environmental friendliness, affordability, sensitivity, specificity, and user friendliness, and the tests should be rapid and robust, equipment free, and deliverable to end users [3,4]. They aim to ensure that POCT is not only accurate and reliable, but also practical for use in various settings, including resource-limited areas.

In the field of genetic testing, sequencing and Polymerase Chain Reaction (PCR) are the most well-known methods. Next-generation sequencing (NGS) has become a cornerstone in modern diagnostics, allowing for the precise identification of genetic disorders and mutations. However, the high cost of NGS limits its widespread adoption, and interpreting NGS data requires specialized expertise to ensure accurate and meaningful results. PCR, which amplifies DNA using two primers complementary to the target sequence, relies on precise temperature changes controlled by sophisticated instruments for the denaturation, annealing, and extension phases. Both NGS and PCR require significant expertise in instrument operation and protocol optimization to achieve accurate and reproducible results, making them unsuitable for POCT applications [5].

To address these limitations, isothermal amplification techniques have gained attention for the development of genetic POCTs. These operate at a constant temperature, eliminating the need for a thermocycler. There are many isothermal amplification methods which can be classified into three categories [6]: exponential amplification [7,8,9,10,11,12,13,14,15], linear amplification [7,16], and in-cascade amplification [17,18].

Examples of isothermal amplification methods include LAMP, NASBA, RPA, SDA, HDA, and MDA. Some are limited to RNA amplification, while others use several enzymes (e.g., NASBA and SDA). The number of primers and operational temperatures also vary depending on the method used, with most techniques operating between 30 °C and 65 °C.

To meet POCT criteria, various devices have been developed to standardize protocols and reduce turnaround times. Examples include the Genie II [19], Twista [20], Nuclisens EasyQ [21], and Samba II [22,23]. Field devices have also been created to eliminate the need for complex equipment, using alternatives such as heating blocks [24,25,26,27], hot plates [28], or heating pads [29] for the heating step.

Moreover, certain devices may incorporate a heating process [30,31,32,33,34,35]. Additionally, to reduce overall costs associated with fluorescence detection, which requires a UV light source, colorimetric detection methods are often preferred for POCT applications. The number of steps should also be significantly decreased, lowering the possibility of contamination and “human” error.

It is important to note that the sample preparation stage, which is often overlooked, can be time-consuming and may require laboratory tools. Some studies use pure DNA samples or do not integrate the preparation stage into the device, which limits their real-life application.

In previous research, we described a device known as SPID (Sampling, Processing, Incubation, Detection), which integrates all stages from sample processing to detection without requiring instruments or electrical power. The entire process is completed in a few simple steps. This device has been validated for detecting antibiotic resistance directly from blood cultures, urine, or rectal swabs [36,37,38]. The detection is performed by a lateral flow immunoassay (LFIA) integrated into the device, and the result can be read visually or with a portable reader.

The objective of this study was to develop a POCT for genetic detection that integrates all steps from sample processing to result interpretation without the need for complex equipment. The aim was also to develop a versatile platform capable of multiplex testing, which is why we are once again combining the SPID device with an LFIA test to form the SPID platform. To achieve this, we employed Loop-Mediated Isothermal Amplification (LAMP), a technique first described by Notomi [14]. Although the high number of primers used in LAMP ensures a high specificity, it also introduces a risk of primer dimer formation, making primer design a critical step [39]. The LAMP technique was adapted to the SPID device to create a simple, rapid process that integrates sample preparation, LAMP reaction, and detection by LFIA. This test is a nucleic acid lateral flow immunoassay (NALFIA) [40].

This study differs from our previous work in that we have included an incubation stage which required the development of a dedicated heating station. In addition, the SPID was modified. In a previous version, the component that punctures the operculum was integrated into the cassette. For this new SPID application, we have designed and produced an adaptor that clips onto the cassette. This new feature, once it has been removed, allows us to deposit the conjugate solution after the sample migration.

For validation of the device, the *E. coli malB* gene, which codes for maltose operon protein B (GenBank sequence: GDB J01648), was chosen as the target for LAMP. The gene is conserved across different *E. coli* lineages but is not common in other Gram-negative bacteria, making it an ideal target for the specific identification of *E. coli* [41]. The new integrated LAMP-based POCT device is perfectly adapted for use in resource-limited settings and enables the rapid, on-site diagnosis of bacterial infections.

## 2. Materials and Methods

### 2.1. Reagents

Unless otherwise mentioned all reagents were from Sigma-Adrich (Saint Quentin Fallavier, France). Bovine serum albumin (BSA, catalog # A7906), casein (catalog # C8654), and streptavidin (catalog #S4762) were from Sigma-Aldrich. Goat anti-mouse (GAM, catalog # 115-005-044) IgG and IgM polyclonal antibodies were from Jackson ImmunoResearch (Baltimore, MD, USA). Monoclonal antibody anti-biotin (Z021, catalog # 03-3700) and Betaine anhydrous (catalog # B24397.22) were from Thermo fisher Scientific (Waltham, MA, USA). Nitrocellulose strips with polystyrene backing (Prima 40, catalog # 10549603), samples (Standard 14, catalog # 8133 2250), and absorbent (CF7, catalog # 8117 2250) pads were from Cytiva (Freiburg, Germany). Culture media: Luria Broth (LB, catalog # L3522) and LB agar (catalog # L2897) were from Sigma-Aldrich. Colloidal gold particles were from NG Biotech Laboratories (Guipry, France). LAMP fluorescent dye (catalog # B1700S), Deoxynucleotide (dNTP) solution mix (catalog # N0447L), magnesium sulfate (MgSO4, catalog # B1003S) solution, and Bst 2.0 Warm Start DNA Polymerase (catalog # M0538L) were from New England Biolabs France (Evry, France).

### 2.2. Solution Preparation

#### 2.2.1. Conjugate Buffer

The conjugate buffer was prepared by diluting 1 g/L of BSA (0.1%), 8.77 g/L of NaCl (0.15 M), 10 g/L of CHAPS (1%), 5 mL/L of tween 20 (0.5%), and 0.01 g/L of NaN_3_ in Tris-HCl 0.1 M pH 8.

#### 2.2.2. LAMP Reaction Solution

To realize the LAMP reaction solution, different stock solutions were prepared:

Stock solution 1:

To prepare this solution, 20 mg of BSA, 200 mg of CHAPS, 3.5 mg of NaCl, 0.1 mL of Tween 20, 3.33 mL of betaine (3 M), 1.6 mL of dNTPs (10 mM), 1.6 mL of MgSO_4_ (100 M), 0.2 mL of Tris-HCl 1 M pH 8.8, and 3.17 mL of H_2_O were mixed.

Stock solution 2:

To prepare this solution, 0.4 mL of KCl 1 M, 0.4 mL of (NH_4_)_2_ SO_4_ 1 M, 40 µL of tween 20, 0.8 mL of Tris-HCl 1 M pH 8.8, and 0.36 mL of H_2_O were mixed.

Stock solution 3:

To prepare this stock solution, 32 µL of FIP (100 µM) and BIP (100 µM), 8 µL of LF (100 µM) and LB (100 µM), 4 µL of F3 (100 µM) and B3 (100 µM), and 12 µL of H_2_O were mixed.

All the above stock solutions were stored at −70 °C. Just before the test, the LAMP reaction solution was prepared by mixing 375 µL of stock solution 1, 75 µL of stock solution 2, 75 µL of stock solution 3, 30 µL of Bst 2.0 Warm Start DNA Polymerase, and 945 µL of H_2_O.

The final composition of the solution was Tris-HCl 25 mM pH 8.8, BIP and FIP 1.6 µM, LB and LF 0.4 µM, B3 and F3 0.2 µM, BSA 0.05%, CHAPS 0.5%, Triton X100 0.1%, Tween 20 0.25%, NaCl 1.5 mM, KCl 10 mM, (NH_4_)_2_ SO_4_ 10 mM, MgSO_4_ 4 mM, dNTP 0.4 mM, betaine 250 mM, and Bst 2.0 Warm Start DNA Polymerase 160 U/mL.

### 2.3. Bacterial Strains

For the validation, 32 bacterial isolates were used to evaluate the sample-to-answer NALFIA test, including a variety of bacterial species: *Escherichia coli*, *Klebsiella pneumoniae*, *Enterobacter cloacae*, *Citrobacter freundii*, *Citrobacter koseri*, *Klebsiella oxytoca*, *Pseudomonas aeruginosa*, and *Proteus mirabilis* (Table S1). These isolates were obtained from Bicêtre Hospital. This collection represented 12 *E. coli* strains and 20 non-*E. coli* strains. For each strain an overnight preculture was realized at 37 °C in LB broth. A 100-fold dilution was then carried out with LB broth and this solution was incubated at 37 °C for 2 h and the turbidity of the culture measured using a densitometer. This tool provides the bacterial concentration in Macfarland units. The solution was then further diluted in LB broth to prepare a 10^8^ UFC/mL suspension.

### 2.4. Colloidal Gold-Labeled Monoclonal Antibody and Streptavidin

This was prepared by adding 25 µL of a 1 mg/mL solution of monoclonal antibody or streptavidin in 20 mM phosphate buffer (pH 7.4) to 2 OD (520 nm) of colloidal gold particles. The final solution was increased to a total of 250 µL with 20 mM borate buffer (pH 9). The mixture was incubated for 1 h at 20 °C, allowing for the adsorption of the proteins onto the surface of the gold particles. This was followed by the addition of 125 µL of a solution containing 10 mM borate buffer (pH 9) and 0.3% casein, which was then centrifuged for 10 min at 10,000× *g*. The supernatant was discarded, and the pellet was suspended in 1 mL of 10 mM borate buffer (pH 9) and 0.1% casein. This was sonicated for a few seconds, and centrifuged for 10 min at 10,000× *g*. The supernatant was again discarded and the pellet suspended in 250 µL of 10 mM borate buffer (pH 9) and 0.1% casein and stored at 4 °C in the dark. The solution obtained corresponds to the conjugate used for the strip tests.

### 2.5. Primer Design

The LAMP primer sets were composed of two inner primers (FIP (F1c-F2) and BIP (B1c-B2)), two outer primers (F3 and B3), and two loop primers (LF and LB) for the detection of the *malB* gene. These primers were designed using the NEB LAMP Primer Design Tool software version 1.4.2 (NEB, Evry, France) (Table 1 and Figure S1). For our test, FIP and BIP were labeled with digoxigenin and biotin, respectively. Primers were purchased from Eurogentec (Seraing, Belgium).

### 2.6. Strip Production

**Figure 1:**
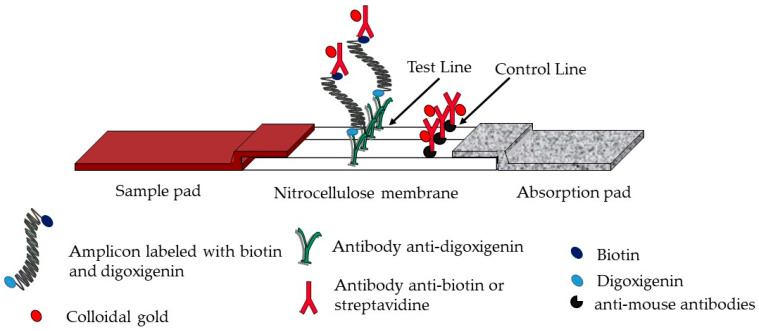
Schematic representation of the test strips. The test strip comprises a sample pad, a nitrocellulose membrane, and an absorption pad. The detection zone uses immobilized anti-digoxigenin antibodies as a test line and anti-mouse antibodies or biotinylated BSA as a control line.

The test strip comprised a sample pad, a nitrocellulose membrane, and an absorption pad, all attached to a backing card. The detection zone used immobilized anti-digoxigenin antibodies (produced by our laboratory) as a test line, and anti-mouse antibodies (goat anti-mouse immunoglobulins, Jackson ImmunoResearch, Baltimore, MD, USA) as a control line (0.8 and 0.5 mg/mL in 50 mM potassium phosphate buffer pH 7.4, respectively), dispensed at 1 μL/cm using an automatic dispenser (DCI-300; Zeta corporation, Gunpo-city, Republic of Korea). After drying for 30 min at 37 °C in an air oven, the absorption pad and the sample pad were glued to the top and bottom edges of the membrane, respectively. The membranes were cut into strips of 5 mm width using an automatic programmable cutter (Guillotine Cutting CM4000; BioDot Irvine, CA, USA). The strips were then placed into a plastic cassette and stored at room temperature with a desiccant. A conjugate pad (Standard 14) could be added between the sample pad and the nitrocellulose. In this case, 10 µL of the conjugate was dried on the conjugate pad. Test strips were inserted into a plastic cassette (Figure 1).

### 2.7. SPID Platform

**Figure 2:**
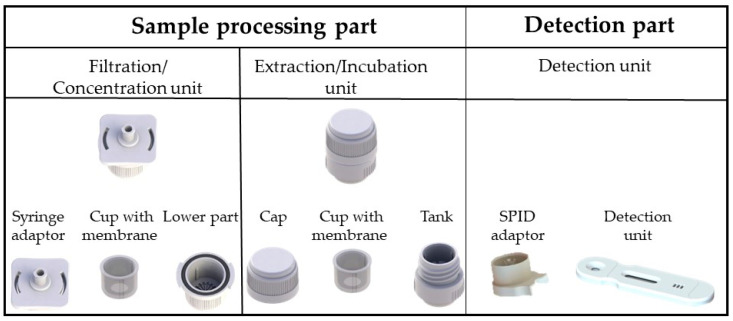
The SPID (Sampling, Processing, Incubation, Detection) platform elements. The SPID platform is composed of two parts: (i) the sample processing part, which includes a filtration/concentration unit consisting of a syringe adaptor, a cup, and a lower part and an extraction unit, consisting of a cap and a tank (SPID Device); and (ii) the detection part, which consists of a SPID adaptor to connect the cassette to the tank, and a plastic cassette integrating a lateral flow immunochromatographic strip.

The SPID platform is a versatile device composed of two parts (Figure 2). The sample processing part includes a filtration/concentration unit consisting of a syringe adaptor, a cup with a 0.45 μm pore size membrane, and a lower unit and an extraction/incubation unit consisting of a cap with a plunger and a tank (SPID device). The detection part consists of a SPID adaptor, which connects the cassette with the tank, and a plastic cassette integrating a lateral flow immunochromatographic strip (Figure 1). The SPID device patented by CEA (EP3528947) is produced by NG Biotech Laboratories.

### 2.8. Heating Station

**Figure 3:**
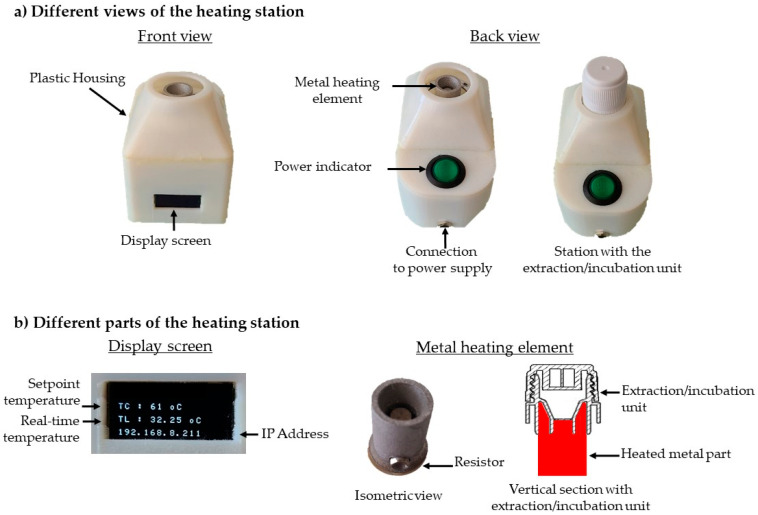
Heating station. The heating station consists of a metal part heated by resistors and adapted to the shape of the tank. The operator has access to the on/off button and a display showing the set temperature, the real-time temperature and the station’s IP address. The IP address can be used to connect to an application to set the desired temperature. All the components are assembled inside a plastic housing.

The heating station comprised a metal heating element, a display screen, a control card, and a Wi-Fi module (Figure 3a). The metal element was heated by an integrated resistor and was specifically designed to fit perfectly around the base of the incubation tank (Figure 3b). All these components were assembled inside a plastic housing. The plastic housing and the metal element were manufactured using 3D printing technology. The display screen (Figure 3b) provides real-time information, including the set temperature, the temperature measured at the metal element, and the station’s IP address. This allows for independent programming and precise temperature control for each heating station.

### 2.9. Test Workflow

**Figure 4:**
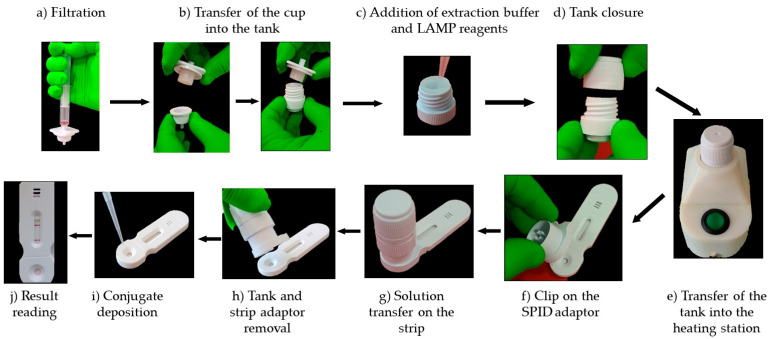
Test workflow. A 1 mL bacterial suspension at 10^8^ CFU/mL is drawn into a syringe and attached to a filtration device. The sample is then pushed through the filter (**a**). The filter cup is subsequently placed in a tank (**b**), and 180 μL of LAMP reaction solution is added (**c**). After sealing the tank (**d**), the system is heated to 63 °C for 30 min (**e**). Once heated, the unit is placed onto a SPID adaptor (**f**,**g**), which punctures the operculum, allowing the liquid to flow onto a strip for migration (**g**). After 5 min, the adapter and reservoir are removed (**h**), and 100 μL of diluted conjugate is applied to the strip (**i**). Results are visually interpreted after 15 and 30 min (**j**).

A 1 mL volume of bacterial suspension at 10^8^ cfu/mL is collected using a syringe that already contains 2 mL of air. The air ensures that the entire liquid volume can be efficiently pushed through the filter. The syringe is screwed onto the filtration device, and the sample is pushed out of the syringe (Figure 4a). The filtration system is then opened by turning it clockwise and the filter cup transferred into the tank by sliding it inside (Figure 4b). A volume of 180 μL of the LAMP reaction solution is added into the filter cup (Figure 4c) and the incubator is closed by screwing down the cap, which forces the sample through the membrane into the tank (Figure 4d). The extraction/incubation unit is positioned on the metal heating element of the station, which heats the solution to 63 °C (Figure 4e). After 30 min, the extraction/incubation unit is clipped on to the SPID adaptor and placed on the cassette (Figure 4f). By pressing firmly downwards, the operculum at the bottom of the tank breaks and allows the liquid to flow onto the strip, thus initiating the migration (Figure 4g). After 5 min, the SPID adaptor and reservoir are removed from the strip (Figure 4h) and 100 µL of conjugate diluted 1/10 in the conjugate solution is applied to the strip (Figure 4i). After 15 and 30 min the results are read visually (Figure 4j). If the test is positive after 15 min, there is no need to read again at 30 min.

## 3. Results

To achieve the study objective of developing a genetic point-of-care test (POCT) that integrates all steps from sample processing to results analysis without the need for complex equipment, several challenges had to be addressed. First, it was necessary to develop a reaction solution capable of extracting DNA from bacteria whilst also being compatible with LAMP as well as LFIA detection. This solution was also required to prevent the non-specific amplifications that can occur during LAMP [42]. Second, a heating station tailored to our device had to be designed and produced. Finally, the LFIA format had to be optimized to detect amplicons in large volumes.

### 3.1. Development of a LAMP Reaction Solution

The LAMP reaction solution was developed based on previous results. The solution combined reagents with an extraction buffer able to extract protein from bacteria and compatible with LFIA [43], and the LAMP reaction buffer has been described in several papers [14,44,45]. The use of a LAMP solution with reagent concentrations identical to those described in the literature did not result in amplification. The concentrations of the various reagents therefore had to be optimized. This optimization led us, for example, to reduce the concentrations of all the reagents used in the extraction buffer as well as the concentration of betaine in the LAMP buffer (Table 2).

The LAMP reaction solution was optimized with the primer set used in the article by Hill et al. [41]. As primers have an impact on the specificity and sensitivity of LAMP [46], we designed a new primer set (Figure S1) and evaluated its performance against the previous set under these optimized conditions using *E. coli* at 10^8^ cfu/mL as positive control and LB broth as negative control. A 1 mL volume of each solution was filtered with the filtration/extraction unit of the SPID device. The cup was then transferred into the tank, and 180 µL of the LAMP reaction solution was added to the cup. The tank was closed, so the liquid filtered to the bottom of the tank. Amplification was then performed using a thermal cycler (CFX Opus 96, Biorad, Hercules, CA, USA). For this, 24.5 µL of the filtrated solution was deposited in a PCR tube with 0.5 µL of LAMP fluorescent dye. The amplification was monitored for 40 min at 63 °C.

**Figure 5:**
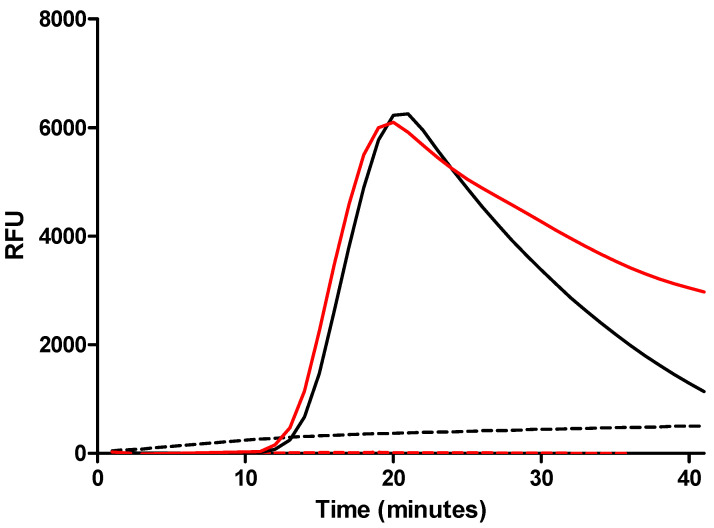
Amplification curves of the *malB* gene using two primer sets. Black curves: amplification using the primer set developed by Hill et al. [41]; red curves: amplification using the set primer design of the current study. The solid lines correspond to the amplification using *E. coli* solution at 10^8^ cfu/mL and the dotted lines correspond to the amplification in LB broth. No amplification was observed for the LB broth. For *E. coli*, amplification began after 10 min of incubation for both primer sets. The amplification curves reached a peak at 20 min and then began to decrease. We observed that the decrease was faster for the primer set used by Hill et al. [41].

The results showed no amplification with LB broth for either primer set (Figure 5). For the *E. coli* solution, amplification began after 10 min of incubation, reaching a peak of fluorescence at 20 min followed by a decrease in fluorescence for both primer sets. These results demonstrated that the reaction solution was able to extract DNA from bacteria and allowed LAMP with the two primers sets. The results also demonstrated that the SPID device could be used to extract DNA from bacteria.

The newly designed primer set provided better coverage across the gene sequence and eliminated overlapping primers, unlike the set used by Hill et al. (Figure S1). We have therefore continued to develop the primer set designed for this study.

The decrease in fluorescence during amplification by LAMP is unusual. To check that this decrease was not linked to a reduction in the quantity of amplicons that could be detected by LFIA, we carried out the same experiment as before, stopping the amplification at different phases and detecting the amplicons present with LFIA. The results obtained (Figure S2) show that there is no link between fluorescence and the signals obtained by LFIA. Indeed, in the last phase of amplification, fluorescence decreased while the LFIA signals continued to increase.

**Figure 6:**
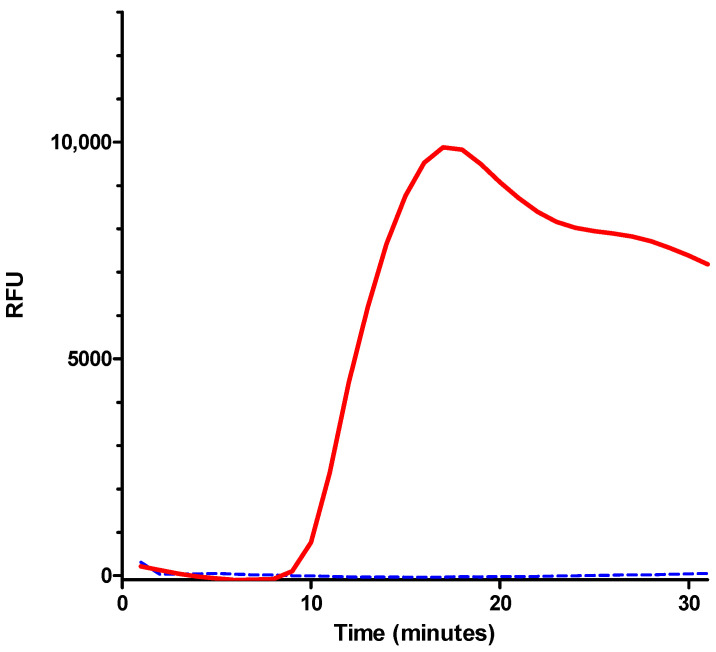
Amplification curves for the *malB* gene. Red curve: amplification of *E. coli*; blue curve: amplification of *C. freundii*. No amplification was observed for *C. freundii*. For *E. coli*, amplification began after 10 min of incubation. The amplification curve reached a peak at 15 min and then began to decrease.

To verify the specificity of this amplification, the previous protocol was applied with 1 mL of bacterial suspensions of *E. coli* or *C. freundii* at 10^8^ cfu/mL. In this experiment the amplification was monitored for 30 min. The results showed that the optimized LAMP reaction solution allowed for the specific detection of *E. coli* (Figure 6).

### 3.2. Detection of Amplicons by LFIA

In the current study, colorimetric methods typically employed to detect gene amplification through color change could not be used due to the opacity of the reservoir. To detect amplicons generated during the LAMP reaction, we employed a nucleic acid lateral flow immunoassay (NALFIA). This approach required two labeled primers: one for capturing the amplicon on the nitrocellulose membrane and another for signal generation (see Section 2). Agarwal et al. [47] previously demonstrated that excess labeled primers, whether unreacted or incorporated into amplicons, could negatively impact the signal intensity of both the test and control lines. Therefore, most NALFIA protocols include a dilution step for the amplification products before they are applied to the strip to ensure a strong signal. However, as our process needed to be fully integrated into a device, diluting the reaction solution post-amplification was not feasible.

To overcome this issue, we compared various strategies for signal generation and the composition of the primer set for amplicon capture on the test line. At the same time, the LAMP reaction solution volume had to be sufficient to effectively extract bacteria from the cup membrane and ensure proper migration along the strip.

#### 3.2.1. Signal Generation

##### Comparison of Streptavidin and Monoclonal Anti-Biotin as Colloidal Gold Conjugates

In our test, the signal was generated via the interaction between the biotin linked to the BIP and a biotin receptor conjugated to colloidal gold. In this experiment, we compared two biotin receptors: streptavidin and a monoclonal antibody (mAb) against biotin.

Volumes of 1 mL of bacterial suspensions containing either *E. coli* or *C. freundii* at 10^8^ cfu/mL were processed as before (filtration, transfer to the tank, measurement of volume of extraction buffer). The tank was subsequently placed into the heating station and incubated at 63 °C for 30 min. Following the incubation, the tank was opened, and 10 µL of either streptavidin or mAb anti-biotin conjugates was added. The tank was reclosed and pressed onto the SPID adaptor positioned on the cassette. After 30 min, the results were read.

**Figure 7:**
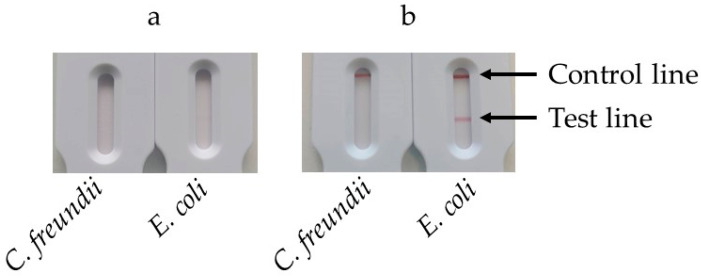
Comparison of streptavidine and mAb anti-biotin as a conjugate. After the extraction/filtration and amplification steps, 10 µL of conjugate was added to the LAMP solution in the tank. The tank was reclosed and pressed onto the SPID adaptor positioned on the cassette. After 30 min the results were read: (**a**) results using streptavidin–colloïdal gold; (**b**) results using mAb anti-biotin–colloïdal gold.

Figure 7a displays the strips obtained using the streptavidin–colloidal gold conjugate. After 30 min of migration, the control line was not visible, and a faint test line appeared on the strip corresponding to *E. coli*. No test line appeared for *C. freundii*. Figure 7b shows the results obtained with the mAb anti-biotin–colloidal gold conjugate. In this case, both the control and the test line exhibited a signal for *E. coli*, whereas only the control exhibited a signal for *C. freundii*.

To ensure comparability, the same strips were used with both conjugates, with a monoclonal anti-digoxigenin on the test line and goat anti-mouse immunoglobulins on the control line. Streptavidin is not recognized by goat anti-mouse immunoglobulins, and it was therefore expected that no signal would appear on the control line when using the streptavidin–colloidal gold conjugate. Despite the high affinity of streptavidin for biotin, the signal observed on the test line was significantly weaker than the one produced by the mAb anti-biotin–colloidal gold conjugate. This weaker signal could have been due to the poor labeling of streptavidin with the colloidal gold or a reduction in its affinity for biotin resulting from adsorption onto the gold nanoparticles. Given its superior performance in visualizing both the control and test lines, the anti-biotin antibody–colloidal gold conjugate was selected for subsequent experiments.

The positive results obtained with the mAb anti-biotin demonstrated that the heating station was functional and allowed for the amplification of the *malB* gene by LAMP in the SPID tank.

##### Comparison of Different Conjugate Deposition Methods

As mentioned above, our aim was to develop a method with a limited number of manipulations. The method used in the previous experiment (one-stage migration), which involved opening the reservoir, depositing the conjugate, and reclosing the reservoir, was not satisfactory. In this experiment, we evaluated alternative conjugate deposition methods to simplify the test workflow.

For the first method, named dried conjugate deposition, the conjugate was dried on a Standard 14 membrane which was inserted between the sample pad and the nitrocellulose membrane. At the end of amplification, the tank was pressed onto the SPID adaptor positioned on the cassette. After 30 min, the results were read (dried conjugate deposition).

For the second method, named two-stage migration, the tank was pressed onto the SPID adaptor at the end of amplification and after 5 min of migration the tank and the SPID adaptor were removed. A volume of 100 µL of diluted conjugate (prepared by mixing 10 µL of conjugate with 90 µL of conjugate buffer) was then applied to the strip and the results were read after 30 min.

For all these methods, 1 mL samples of bacterial suspensions containing either *E. coli* or *C. freundii* at 10^8^ cfu/mL were used.

**Figure 8:**
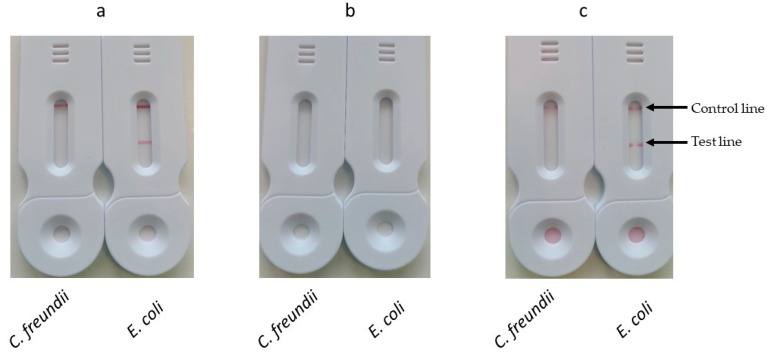
Comparison of different methods for the conjugate deposition. (**a**) One-stage deposition. The tank was opened and 10 µL of either streptavidin or mAb anti-biotin conjugates were added. The tank was reclosed and pressed onto the SPID adaptor positioned on the cassette. (**b**) Dried conjugate deposition. The conjugate was dried on a Standard 14 membrane which was inserted between the sample pad and the nitrocellulose membrane. The tank was pressed onto the SPID adaptor positioned on the cassette. (**c**) Two-stage deposition. The tank was pressed onto the SPID adaptor and after 5 min of migration the tank and the SPID adaptor were removed. A volume of 100 µL of diluted conjugate (prepared by mixing 10 µL of conjugate with 90 µL of conjugate buffer) was then applied to the strip. For all these conditions, the results were read after 30 min.

The results (Figure 8) showed that both one-stage and two-stage depositions allowed for the visualization of test and control lines, while dried conjugate deposition failed to produce visible test or control lines.

The dried conjugate deposition implies the resolubilization of the dry conjugate by the sample. This probably leads to a non-homogeneous mixing of the sample and conjugate. As a result, the conjugate becomes more concentrated at the top of the migration, while the final microliters of the sample lose access to the conjugate due to its washout from the conjugate pad. In contrast, the one-step method ensures a perfectly homogeneous mixture of the sample and conjugate.

The lack of signal for the dried conjugate deposition may also be explained by the shorter contact time between the conjugate and the sample. In this method, the contact time is limited to the migration period between the conjugate pad and the test line. In contrast, the one-stage deposition method allows the conjugate to interact with the sample before being applied to the strip, providing a longer contact time.

The signals obtained at the test and control lines for the one-stage and two-stage depositions were identical. The second method offers two advantages: (1) it is simpler and requires less handling, and (2) it eliminates excess BIP–biotin primers during the migration of the amplification solution. As a result, the conjugate will specifically react only with the biotin on the amplicons immobilized at the test line.

Based on the results of this experiment, the two-step deposition method was chosen for further studies.

#### 3.2.2. Capture

To evaluate the effect of the FIP–digoxigenin primer concentration on signal intensity at the test line, primers with varying concentrations of FIP–digoxigenin were used during amplification. To ensure efficient amplification, a minimum primer concentration was necessary. Unlabeled FIP was therefore added to maintain the overall FIP concentration in the different mixes (Table 3).

**Figure 9:**
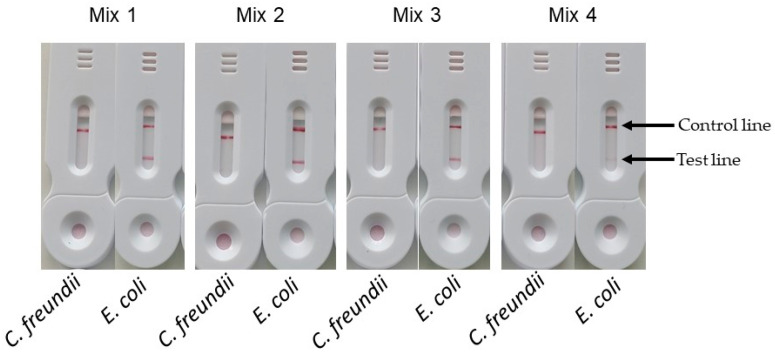
Comparison of different concentrations of FIP–digoxigenin. In this experiment all the primer mixes contained 1.6 µM of BIP–biotin, 0.2 µM of B3 and F3, and 0.4 µM of LB and LF. Mix 1 contained 1.6 µM of FIP–digoxingenin; Mix 2 contained 0.8 µM of FIP–digoxingenin and 0.8 µM of FIP; Mix 3 contained 0.4 µM of FIP–digoxingenin and 1.2 µM of FIP; and mix 4 contained 0.2 µM of FIP–digoxingenin and 1.4 µM of FIP.

Figure 9 shows the results obtained with the different primer mixes using 1 mL of bacterial suspensions containing either *E. coli* or *C. freundii* at 10^8^ cfu/mL. The signals obtained on the test line for mixes 1, 2, and 3 were comparable, while the test line signal for mix 4 was significantly weaker. Contrary to previous reports, our tests showed that the highest concentrations of labeled primers did not result in a decrease in signal intensity at the test line. These results also indicate that achieving an optimal signal requires a sufficient proportion of labeled primer, which in this case was over 25%.

Given the high cost of FIP–digoxigenin primers and our aim to minimize the cost per test, we selected a concentration of 0.4 µM FIP–digoxigenin combined with 1.2 µM FIP (mix 3) for our subsequent experiments.

### 3.3. Limit of Detection

The limit of detection was evaluated by filtering 1 mL *E. coli* bacterial suspensions at concentrations of 10^8^, 10^7^, 10^6^, and 0 cfu/mL. The test was conducted under the previously optimized conditions. This experiment was repeated three times in monoplicate with different *E. coli* solutions, a different Bst, and on different days (Figure S3).

**Figure 10:**
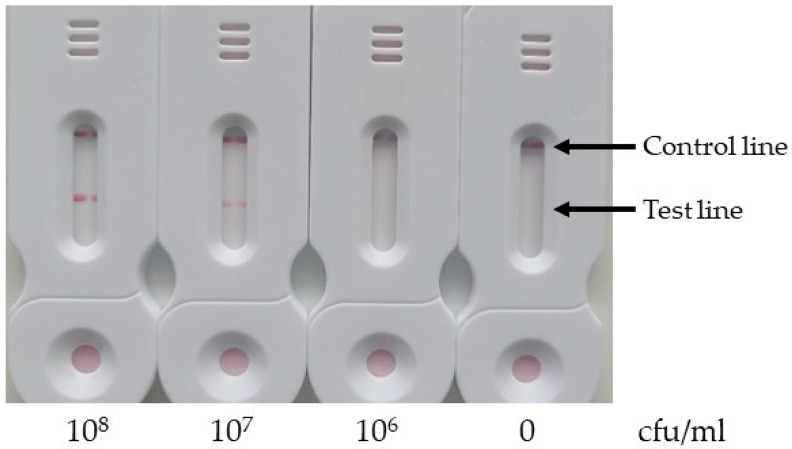
Evaluation of the limit of detection. Different concentrations of *E. coli* were tested for 30 min of amplification at 63 °C. A test line was visible for the 10^8^ and 10^7^ cfu/mL concentrations.

As shown in Figure 10, a signal was detected on the test line for concentrations of 10^8^ and 10^7^ cfu/mL, but not for 10^6^ cfu/mL. The limit of detection of our test is therefore between 10^7^ and 10^6^ cfu/mL.

Several factors may contribute to the high detection limit observed: (1) the DNA extraction may not have been optimal; (2) the LAMP reaction solution, while not completely inhibiting the activity of the Bst2 enzyme, may reduce it; (3) the 30 min amplification time, chosen for rapid testing, may be insufficient to achieve optimal sensitivity.

Future research will focus on optimizing each of these parameters to improve the system’s performance.

### 3.4. Validation

Our test for the *E. coli* identification was validated using 32 bacterial isolates, including 12 *E. coli* strains and 20 *non-E*. *coli* strains. All *E. coli* strains were accurately identified. However, among the *non-E. coli* strains, three *C. freundii* were incorrectly identified as *E. coli*, while the remaining strains were correctly classified as *non-E. coli* (Table 4). The absence of the *malB* gene in the three *C. freundii* strains giving a positive signal was verified by PCR. These false positive results were probably due to non-specific amplifications during the LAMP.

This validation enabled us to determine that the test’s sensitivity was 100% and its specificity was 85%.

## 4. Discussion

The aim of this project was to develop a field-ready platform that integrates all stages of genetic analysis, from sample processing to results analysis, without requiring complex equipment. This platform could be deployed in resource-limited settings or used in time-sensitive applications such as infectious disease outbreaks, environmental monitoring, or point-of-care diagnostics.

Among the available isothermal amplification techniques, we focused on LAMP due to its rapid amplification, compatibility with simple visual detection methods, and resistance to inhibitors commonly present in samples. The point-of-care test combined LAMP with the SPID platform. The latter is already used to filter, concentrate, and extract proteins from bacterial matrices directly in clinical samples. The SPID device was combined with LFIA technology for the genetic detection. Indeed, even if this technology extends the duration of the test, it allows for multiplex detection.

Several challenges emerged during development, including the (1) creation of a multifunctional buffer capable of supporting bacterial DNA extraction, LAMP, and detection by LFIA; (2) the detection of amplicons with LFIA without the need for sample dilution; and (3) the development of a heating station to maintain a constant temperature in the SPID tank during amplification.

Our study yielded promising results, including the development of a novel reaction solution that integrates DNA extraction, LAMP, and LFIA detection. During the development process we constantly had to find a balance between these three functions. For example, CHAPS is essential for bacterial lysis, but at too high a concentration it inhibits amplification. Betaine reduces non-specific amplification, but when combined with CHAPS also inhibits amplification. Finally, NaCl, which reduces non-specific binding during detection by LFIA, interferes with amplification.

A comparison of two detection systems (streptavidin and a monoclonal antibody against biotin) showed a clear advantage of the monoclonal antibody system for an improved visualization of both the test and control lines. This finding was unexpected as streptavidin is known for its high affinity for biotin. We propose two hypotheses to explain this result: (1) the labeling of streptavidin with colloidal gold nanoparticles may be less efficient than that of antibodies; and (2) the adsorption of streptavidin onto colloidal gold may cause conformational changes, reducing its affinity for biotin.

Contrary to previous reports indicating that an excess of labeled primers may inhibit the signal in NALFIA, our results showed a signal for a labeled monoclonal antibody even when the entire sample was applied to the strip in a one-step deposition. Despite this, we used two of the strategies evaluated to counteract this inhibition. Indeed, the two-step deposition protocol was a good compromise between the dried tracer and one-stage deposition. The disadvantage of one-stage deposition is that the reservoir has to be opened and reclosed after amplification, increasing the risk of contamination and introducing additional handling steps. The dried tracer method, while simplifying the process, failed to produce detectable signals on the test strips.

A comparative study of different FIP–digoxigenin concentrations revealed that using a 4-fold lower concentration had no effect on signal intensity. Given the higher cost of FIP–digoxigenin compared to unmodified FIP primers, we opted for this concentration to keep the cost per test as low as possible. Currently, the cost per test is approximately EUR 12–13. However, with large-scale production, this cost is expected to decrease significantly.

The optimized protocol was validated on 32 samples for the identification of *E. coli*. The test demonstrated 100% sensitivity and 85% specificity, confirming that our SPID platform, in combination with the handheld heating station, provides an effective sample-to-answer genetic test suitable for field applications. The limit of detection of our test was quite high, but will be sufficient for some applications such as genetic detection in blood culture samples.

In this study, we observed a difference in the evolution of the fluorescent signal and the signals obtained by LFIA during amplification. The fluorescent signal was generated by the binding of fluorescent with double-strand DNA, whereas the LFIA signal was due to the presence of two probes on the amplicons. Unlike PCR, which produces double-stranded amplicons of the same size, LAMP generates amplicons of different sizes with different structures (double-stranded, single-stranded, and single-loop) [48]. In addition, for PCR each of the probes is located on a different strand of DNA, whereas for LAMP the two probes can be located on the same strand. Under our amplification conditions, there could, therefore, be an increase in the production of amplicons with single-stranded structures, which would result in a decrease in fluorescence but not in the signal obtained by LFIA.

To the best of our knowledge, only one sample-to-answer platform using LFIA technology and integrating all the stages of a genetic test has been published [49]. The other platforms use colorimetric or fluorescent detection (Table 5). These latter methods do not allow for multiplex detection, and the interpretation of their color changes or fluorescence is not always straightforward. The LFIA technology platform uses five syringes containing different reagents and five three-way valves. The syringes and the three-way valves have to be changed between each sample. In addition, the test requires the use of three different temperatures, which implies the development of a dedicated instrument to manage the different temperatures and the operation of the different valves. In comparison, the set-up for our platform was much simpler and the test only required a basic portable heating station. That is why our test can be considered to be a sample-to-answer POCT.

Although several POCTs utilizing LAMP and LFIA technologies have been described (Table 6), none are sample-to-result platforms because they require a preliminary extraction step and/or a dilution step for the amplicons before the LFIA test. It should be noted that, despite our test incorporating all the essential stages required for its execution, its duration remains comparable to that of POCTs utilizing LFIAs.

The present study represents a proof-of-concept study and future work will have to be carried out in order to (1) validate the test in more complex matrices such as urine, blood cultures, environmental, and veterinary samples and (2) improve the limit of detection to extend the area of test application. Moreover, the potential to expand the SPID platform for the simultaneous detection of both proteins and genes will be evaluated.

## Figures and Tables

**Table 1:** Characteristics of the primers used for the *malB* gene amplification.

Name	Sequence	5′pos	3′pos	Length	Tm
F3	GGTGTCGATGACAGGTTGTT	55	74	20	59.71
B3	CCGTTTCTCACCGATGAACA	269	288	20	59.71
F2	CAAAGGGAGAAGGGCATGG	76	94	19	59.87
F1c	GATACCACGACCTCGCCCCA	127	146	20	65.62
B2	TCTCACGCCCGGCAATCA	233	250	18	60.75
B1c	ATTCGTGGTGTTTGTCGGACCG	180	201	22	65.16
FIP (F1c-F2)	GATACCACGACCTCGCCCCACAAAGGGAGAAGGGCATGG			39	
BIP (B1c-B2)	ATTCGTGGTGTTTGTCGGACCGTCTCACGCCCGGCAATCA			40	
LF	CGTTACATTTTGCAGCTGTACGC	98	120	23	64.92
LB	GGCTGCGGTAAATCGACTTTACT	205	227	23	64.95

**Table 2:** Compositions of the extraction and LAMP reaction buffers and LAMP reaction solution.

	Reagents	Original Buffer Composition			LAMP Reaction Solution
Extraction buffer (Ref. [42])	Tris-HCl pH 8	100 mM			-
	NaCl	0.15 M NaCl			0.0015 M
	BSA	0.1%			0.05%
	Tween 20	0.5%			0.25%
	CHAPS	1%			0.5%
		Ref. [14]	Ref. [43]	Ref. [44]	
LAMP reaction buffers	Tris-HCl pH 8.8	20 mM	20 mM	20 mM	25 mM
	FIP/BIP	0.8 µM	1.6 µM	2 µM	1.6 µM
	F3/B3	0.2 µM	0.2 µM	0.2 µM	0.4 µM
	LB/LF	-	-	1 µM	0.2 µM
	dNTP	1.6 mM	5.6 mM	1.4 mM	0.4 mM
	KCl	10 mM	10 mM	10 mM	10 mM
	(NH_4_)_2_SO_4_	10 mM	10 mM	10 mM	10 mM
	MgSO_4_	4 mM	8 mM	8 mM	4 mM
	Triton X-100	0.1%	-	-	0.1%
	Betain	1 M	0.8 M	0.8 M	0.25 M
	Bst	320 U/mL	320 U/mL	320 U/mL	160 U/mL
	Tween-20	-	-	0.1%	-

**Table 3:** Composition of the different primer mixes.

Primer	Mix 1	Mix 2	Mix 3	Mix 4
BIP–biotin	1.6 µM	1.6 µM	1.6 µM	1.6 µM
FIP	-	0.8 µM	1.2 µM	1.4 µM
FIP–digoxigenin	1.6 µM	0.8 µM	0.4 µM	0.2 µM
B3	0.2 µM	0.2 µM	0.2 µM	0.2 µM
F3	0.2 µM	0.2 µM	0.2 µM	0.2 µM
LB	0.4 µM	0.4 µM	0.4 µM	0.4 µM
LF	0.4 µM	0.4 µM	0.4 µM	0.4 µM

**Table 4:** Validation results.

Strains	Numbre of Isolates	Positive Results	Negative Results
*E. coli*	12	12	0
*K. pneumoniae*	10	0	10
*E. cloacae*	1	0	1
*C. freundii*	4	3	1
*C. koseri*	1	0	1
*K. oxytoca*	1	0	1
*P. aeruginosa*	2	0	2
*P. mirabilis*	1	0	1

**Table 5:** Sample-to-answer platform using LAMP.

Extraction	Amplification	Detection	Comments	Total Time	References
Lysis by heat	30 min	Colorimetric	Microfluidic device with four valves and two chambers	40 min	[50]
Lysis by heat	30–40 min	Colorimetric	Lyophilized reagent Detection by change in color Incubation thermos	55 min	[51]
Lysis buffer	35 min	Colorimetric	Microfluidic device for sample processing Heating with a coffee mug	50 min	[52]
Lysis buffer	15–20 min	Colorimetric	Use of a microfluidic chip and portable equipment	40 min	[53]
Lysis buffer and mechanical grinding	30 min + 4 − 10 min	Fluorescence	Extraction carried out using an independent platform Several filtration and washing steps during extraction	40 min + extraction	[54]
Lysis by heat	30 min	LFIA	Need different temperatures Used five single syringes and five three-way valves Dedicated instrument for the automation	40 min (no indication for the detection step)	[49]
Lysis buffer	30 min	LFIA		60–75 min	Our study

**Table 6:** POCTs using LAMP and LFIA.

Extraction	Amplification Time	Dilution Before LFIA	Comments	Total Time	Reference
No	60 min	No	Use of purified DNA Use of portable equipment	75 min	[55]
Yes	50 min	Yes	Use of heat block Multiplex detection	60 min	[56]
No	60 min	No	Use of an extraction kit Used of a microfluidic chip	Less than 120	[57]
No	30 min	Yes	Use of an extraction kit Multiplex detection	40 min	[58]

## Data Availability

The original contributions presented in the study are included in the article/Supplementary Materials, further inquiries can be directed to the corresponding author.

## Supplementary Materials

The following are available online at https://www.mdpi.com/article/10.3390/bios14120609/s1, Figure S1: The sequence of the *malB gene* with the different primer sets used. (A) The sequence of *malB* gene with the position of the different primers used in the publication of Hill, J. et al. The red rectangle indicates the position of the overlap between primers F2 and LF. (B) The sequence of *malB* gene with the position of the different primers designed in this study. Figure S2: Amplicon detection by LFIA at different stages of the amplification curve of the *malB* gene. 1 mL of *E. coli* at 10^8^ cfu/ml was filtered with the filtration/extraction unit of the SPID. The cup was then transferred into the tank, and 180 µL of LAMP reaction solution were added to the cup. The tank was closed, so the liquid filtered down to the bottom of the tank. Amplification was then performed using a thermal cycler (CFX Opus 96, Biorad, Hercules, CA, USA). For this, 24.5 µL of the filtrated solution were deposited in a PCR tube with 0.5 µL of LAMP fluorescent dye. The amplification at 63 °C was stopped at different times. 10 µL of the solution was mixed with 10 µL of mAb ant-biotin labeled with colloidal gold and 80 µL of the conjugate buffer. The mixture was deposited on the LFIA test and the result was read after 15 min. The graph represents the amplification curve and the numbers indicate the stages of the different amplicon detections. Below the graph we can see the results of the amplicon detection with LFIA at the different stages. Figure S3: Evaluation of the limit of detection. Different concentrations of *E. coli* were tested for 30 min amplification at 63 °C. The results were read after 30 min. A and B: Experiments carried out on the same day but with a different Bst enzyme. C: Experiment carried out on another day. Table S1: List of bacterial isolates used in this study.
