# Supplementary material for: Establishment of Sample-to-Answer Loop-Mediated Isothermal Amplification-Based Nucleic Acid Testing Using the Sampling, Processing, Incubation, Detection and Lateral Flow Immunoassay Platforms
Source: Biosensors (Basel). 2024 Dec 13;14(12):609. doi: 10.3390/bios14120609 (PMC11674888; doi:10.3390/bios14120609)
Supplement: Supplementary file 1 [file biosensors-14-00609-s001.zip › biosensors-3268707-supplementary.pdf]

A)

```
                  GCCATCT CCTGATGACG C(F3)  AGC CCATCATGAA
  1 GTGGAGGATT TAAGCCATCT CCTGATGACG CATAGTCAGC CCATCATGAA
    CACCTCCTAA ATTCGGTAGA GGACTACTGC GTATCAGTCG GGTAGTACTT

    TGTTGGT(F2)
 51 TGTTGGTGTC GATGACAGGT TGTTACAAAG GGAGAAGGGC ATGGCGAGCG
    ACAACCACAG CTACTGTCCA ACAATGTTTC CCTCTTCCCG TACCGCTCGC
          ACAG CTACTGTCCA ACAATGTTTC(LF)           CGCTCGC

                               CTGGG GCGAGGTCGT GGTAT(B1c)
101 TACAGCTGCA AAATGTAACG AAAGCCTGGG GCGAGGTCGT GGTATCGAAA
    ATGTCGACGT TTTACATTGC TTTCGGACCC CGCTCCAGCA CCATAGCTTT
    ATGTCGACGT TTTAC(F1c)

       ATCAATC TCGATATCCA TGAAGGTG(LB)
151 GATATCAATC TCGATATCCA TGAAGGTGAA TTCGTGGTGT TTGTCGGACC
    CTATAGTTAG AGCTATAGGT ACTTCCACTT AAGCACCACA AACAGCCTGG
                                  TT AAGCACCACA AACAGCCT(B2)
                                                         G
201 GTCTGGCTGC GGTAAATCGA CTTTACTGCG CATGATTGCC GGGCTTGAGA
    CAGACCGACG CCATTTAGCT GAAATGACGC GTACTAACGG CCCGAACTCT
    CAGACCGACG CCATTTA(B3)

251 CGATCACCAG CGGCGACCTG TTCATCGGTG AGAAACGGAT GAATGACACT
    GCTAGTGGTC GCCGCTGGAC AAGTAGCCAC TCTTTGCCTA CTTACTGTGA

301 CCGCCAGCAG AACGCGGCGT TGGTATGGTG TTTC
    GGCGGTCGTC TTGCGCCGCA ACCATACCAC AAAG
```

B)

```
  1 GTGGAGGATT TAAGCCATCT CCTGATGACG CATAGTCAGC CCATCATGAA
    CACCTCCTAA ATTCGGTAGA GGACTACTGC GTATCAGTCG GGTAGTACTT

                               CAAAG GGAGAAGGGC ATGG(F2)
        GGTGTC GATGACAGGT TGTT(F3)
 51 TGTTGGTGTC GATGACAGGT TGTTACAAAG GGAGAAGGGC ATGGCGAGCG
    ACAACCACAG CTACTGTCCA ACAATGTTTC CCTCTTCCCG TACCGCTCGC
                                                       CGC

101 TACAGCTGCA AAATGTAACG AAAGCCTGGG GCGAGGTCGT GGTATCGAAA
    ATGTCGACGT TTTACATTGC TTTCGGACCC CGCTCCAGCA CCATAGCTTT
    ATGTCGACGT TTTACATTGC(LF)   ACCC CGCTCCAGCA CCATAG(F1c)

                                   A TTCGTGGTGT TTGTCGGACC
151 GATATCAATC TCGATATCCA TGAAGGTGAA TTCGTGGTGT TTGTCGGACC
    CTATAGTTAG AGCTATAGGT ACTTCCACTT AAGCACCACA AACAGCCTGG

    G(B1c)     GGCTGC GGTAAATCGA CTTTACT(LB)
201 GTCTGGCTGC GGTAAATCGA CTTTACTGCG CATGATTGCC GGGCTTGAGA
    CAGACCGACG CCATTTAGCT GAAATGACGC GTACTAACGG CCCGAACTCT
                                       ACTAACGG CCCGAACTCT(B2)

251 CGATCACCAG CGGCGACCTG TTCATCGGTG AGAAACGGAT GAATGACACT
    GCTAGTGGTC GCCGCTGGAC AAGTAGCCAC TCTTTGCCTA CTTACTGTGA
                       AC AAGTAGCCAC TCTTTGCC (B3)

301 CCGCCAGCAG AACGCGGCGT TGGTATGGTG TTTC
    GGCGGTCGTC TTGCGCCGCA ACCATACCAC AAAG
```

Figure S1: The sequence of the *malB gene* with the different primer sets used. **A)** The sequence of *malB* gene with the position of the different primers used in the publication of Hill, J.et al. The red rectangle indicates the position of the overlap between primers F2 and LF. **B)** The sequence of *malB* gene with the position of the different primers designed in this study.

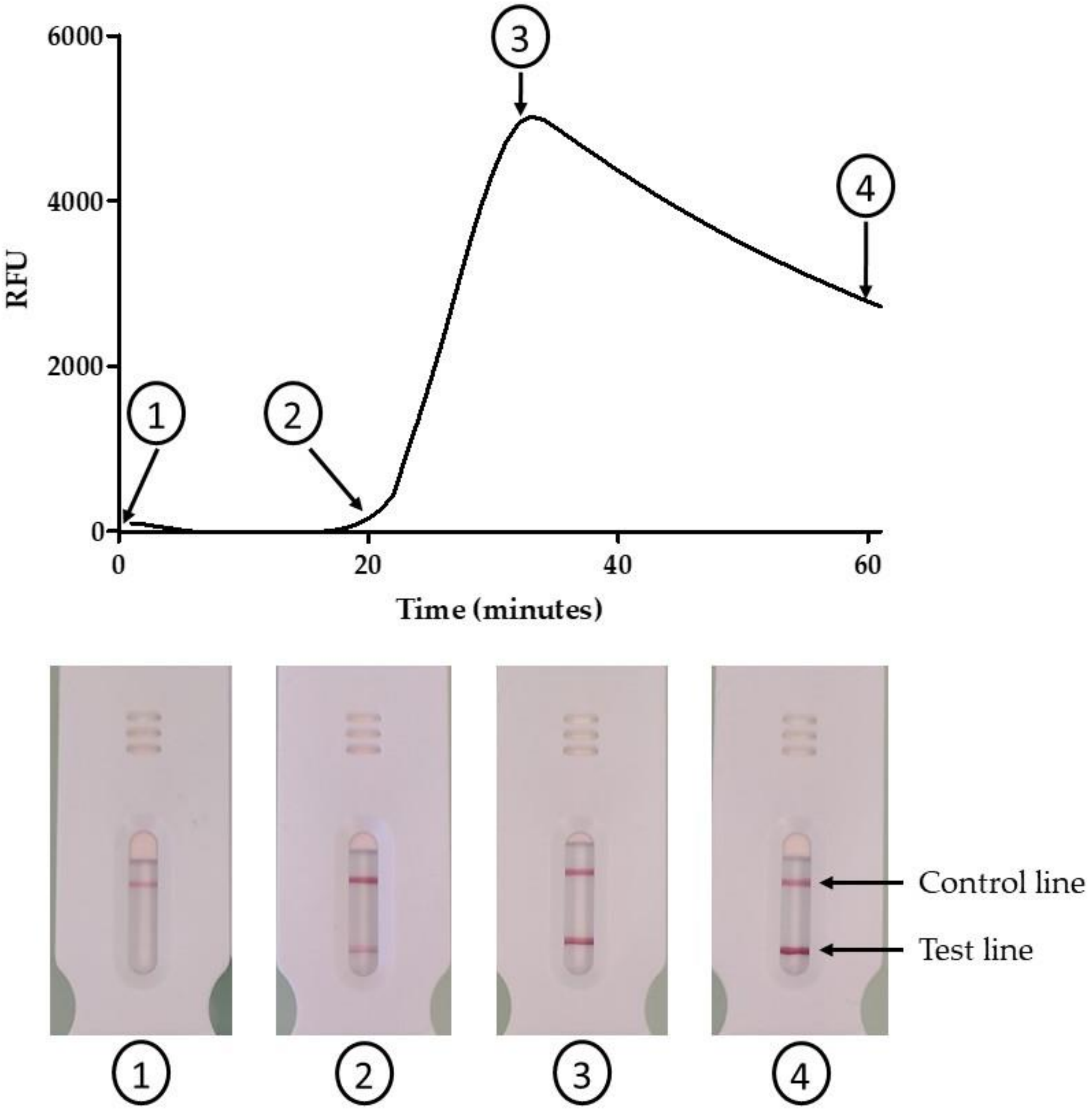

Figure S2: Amplicon detection by LFIA at different stages of the amplification curve of the *malB* gene. 1 ml of E. coli at 10^8 cfu/ml was filtered with the filtration/extraction unit of the SPID. The cup was then transferred into the tank, and 180 µL of LAMP reaction solution were added to the cup. The tank was closed, so the liquid filtered down to the bottom of the tank. Amplification was then performed using a thermal cycler (CFX Opus 96, Biorad, Hercules, USA). For this, 24.5 µL of the filtrated solution were deposited in a PCR tube with 0.5 µL of LAMP fluorescent dye. The amplification at 63°C was stopped at different times. 10 µl of the solution was mixed with 10 µl of mAb ant-biotin labeled with colloidal gold and 80 µl of the conjugate buffer. The mixture was deposited on the LFIA test and the result was read after 15 minutes. The graph represents the amplification curve and the numbers indicate the stages of the different amplicon detections. Below the graph we can see the results of the amplicon detection with LFIA at the different stages.

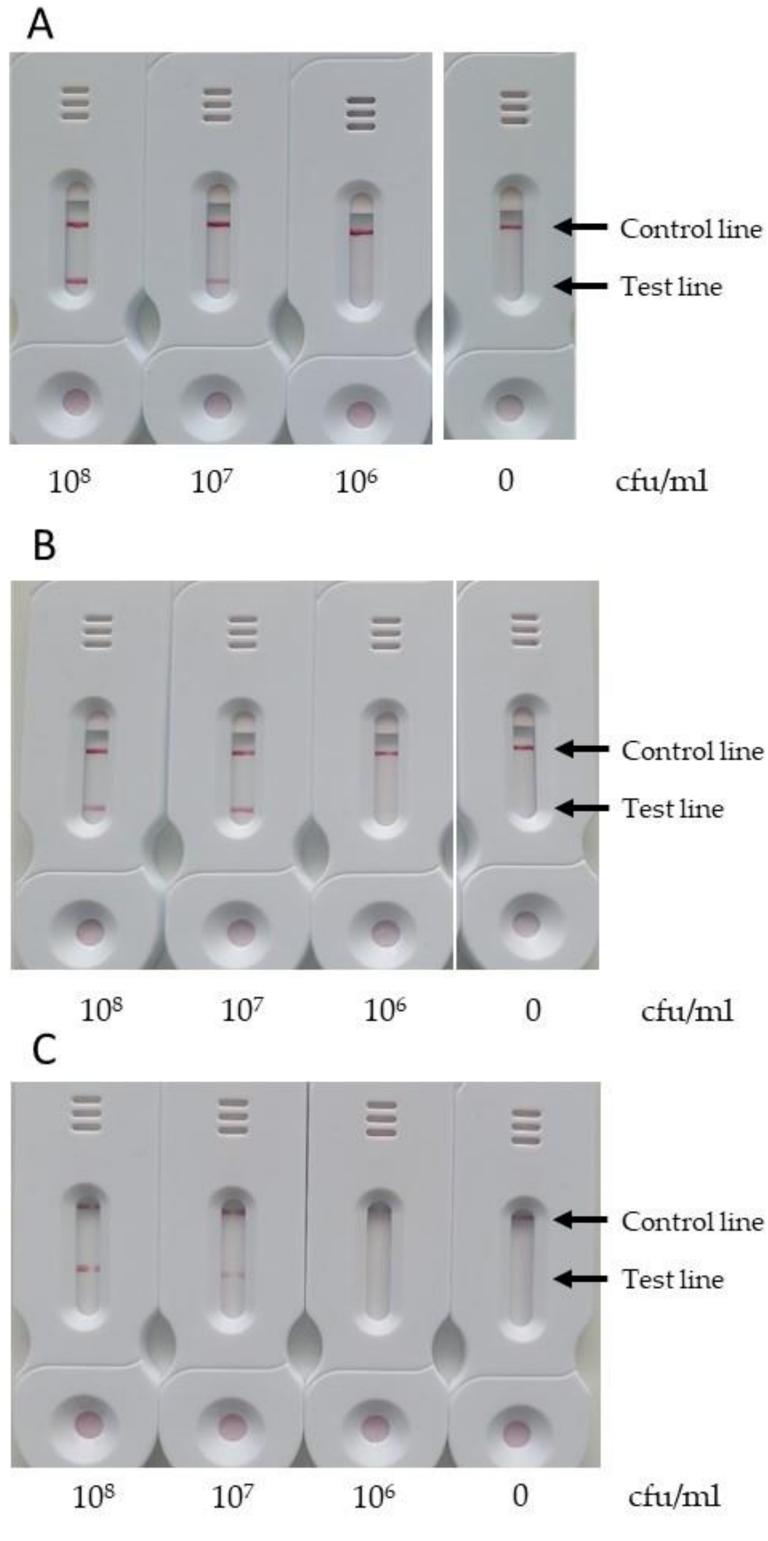

Figure S3: Evaluation of the limit of detection. Different concentrations of *E. coli* were tested for 30 minutes amplification at 63°C. The results were read after 30 minutes. **A** and **B**: Experiments carried out on the same day but with a different Bst enzyme. **C**: Experiment carried out on another day.

Table S1: List of bacterial isolates used in this study

Isolate #	Isolate name	Organism
1	2009-47	*C. freundii*
2	2008-85	*E. coli*
3	EcM-8	*E. coli*
4	210135	*C. freundii*
5	2009-85	*K. oxytoca*
6	G1R4	*K. pneumoniae*
7	19/08/15	*K. pneumoniae*
8	2010-73	*E. coli*
9	2008-87	*E. coli*
10	A3O31	*E. coli*
11	2010-231	*E. coli*
12	2011-11C	*P. mirabilis*
13	31MC1	*E. coli*
14	M271	*E. coli*
15	EcM-8	*E. coli*
16	ST131	*E. coli*
17	ST157	*K. pneumoniae*
18	1F9	*K. pneumoniae*
19	3B1	*K. pneumoniae*
20	D3R10	*K. pneumoniae*
21	1A10	*E. coli*
22	1B1	*K. pneumoniae*
23	1A6	*E. coli*
24	2D1	*C. freundii*
25	O63J6	*P. aeruginosa*
26	2B9	*C. koseri*
27	2B6	*E. cloacae*
28	2A9	*K. pneumoniae*
29	140H2	*C. freundii*
30	I1DR8	*K. pneumoniae*
31	J1R8	*K. pneumoniae*
32	F1O26	*P. aeruginosa*
